# The complete mitochondrial genome of the subsocial cockroach *Nauphoeta cinerea* and phylogenomic analyses of Blattodea mitogenomes suggest reclassification of superfamilies

**DOI:** 10.1080/23802359.2017.1285207

**Published:** 2017-02-06

**Authors:** Ana Teresa Nogueira Dumans, Danielle Bertino Grimaldi, Carolina Furtado, Ednildo de Alcantara Machado, Francisco Prosdocimi

**Affiliations:** a Laboratório de Genômica e Biodiversidade, Instituto de Bioquímica Médica Leopoldo de Meis, Universidade Federal do Rio de Janeiro, Rio de Janeiro, Brazil;; b Departamento de Genética e Biologia Molecular, Universidade Federal do Estado do Rio de Janeiro, Rio de Janeiro, Brazil;; c Laboratório de Bioquímica de Insetos e Parasitos, Instituto de Biofísica Carlos Chagas Filho, Universidade Federal do Rio de Janeiro, Rio de Janeiro, Brazil;; d Departamento de Genética, Instituto Nacional de Cancer, Rio de Janeiro, Brazil

**Keywords:** *Nauphoeta cinerea*, mitochondrial genome, phylogenomics, Blattodea, Corydioidea

## Abstract

The genome of the subsocial cockroach *Nauphoeta cinerea* was partially sequenced in one-twelfth of an Illumina HiSeq lane. The mitochondrial genome was assembled using MIRA software, yielding a circular molecule of 15,923 bp in length and deposited in GenBank under the accession number KY212743. As expected, the mitogenome contained 13 protein-coding genes, 22 transfer tRNAs and 2 ribosomal RNAs. The molecule was assembled using 35,163 sequencing reads of 120 bp each, resulting in ∼286.9× coverage of uniformly distributed reads along the genome. All the 6 complete mitochondrial genomes available for the roaches from the superfamily Blaberoidea were downloaded and compared with the mitogenome of *N. cinerea*. We also downloaded complete mitochondrial genomes from the superfamily Blattoidea, including 6 mitochondrial genomes of Termitoidae, 2 mitogenomes of Cryptocercoidae and 3 from Blattoidae. A supermatrix dataset presenting the concatenated alignment of all mitochondrial genes was used as input for a maximum likelihood phylogeny. The phylogenomic tree obtained was consistent for most clades, with a relevant exception in the position of the Corydioidea species *E. sinensis*. Mitochondrial gene information suggests that superfamily Corydioidea should be classified as a clade inside Blattoidea. Nuclear markers and other Corydioidea mitogenomes should be studied to confirm the evolutionary relationships of Blattodea superfamilies.

Cockroaches are an extremely diverse clade of insects with ∼4000 species described (Velez et al. 2006). Roaches can be found in most terrestrial habitats and present adaptations to survive in extreme conditions (Bohn et al. 2010). *Nauphoeta cinerea* is known as the gray cockroach and it is the sole representative of its genus. It is a Blaberidae, ovoviviparous cockroach. *N. cinerea* is a model species for sexual selection (Bouchebti et al. 2016) and correlations between metabolic rate and fitness studies (Schimpf et al. 2012). The sample sequenced here was obtained from a Brazilian enterprise that sells *N. cinerea* specimens for reptile feeding, though the providers could not specify the precise location on which the original specimens were collected. Following morphological identification, the sample was kept at −20 °C under the voucher number NAU_001 at Genomics and Biodiversity Laboratory.

The partial genome of *N. cinerea* was sequenced in Illumina HiSeq and produced 17,582,642 paired-end sequences of 120 bp, producing 2.1Gb of DNA in fastQ format. The entire set of reads was input into a *de novo* assembly using MIRA (Chevreux et al. 1999) and mitoMaker (http://sourceforge.net/projects/mitomaker/). Both software produced nearly complete versions of the mitochondrial genome when compared to the mitogenome of *Gromphadorhina portentosa*. We found contigs that extended those parts and used them to manually produce a complete version of the *N. cinerea* mitogenome. This version was used as a backbone for a reference-based assembly by MIRA.

We therefore produced a circularized, trustworthy version of the gray cockroach mitogenome using 35.163 sequencing reads. The final version was confirmed by manual inspection of uniformly distributed reads along the genome with an average coverage of 286× according to Tablet (Milne et al. 2013). The complete mitogenome was found to be 15,923 bp in length and was deposited in GenBank under the accession number KY212743. Automatic annotation was performed using *geneCheker* script from mitoMaker package (Schomaker and Prosdocimi, unpublished) followed by manual curation using Artemis (Carver et al. 2012).

We downloaded 18 complete mitochondrial genomes from the order Blattodea, including all mitogenomes available for the superfamily Blaberoidea, 6 of Termitoidae, 2 of Cryptocercoidae, 3 from Blattoidae and 1 from Corydioidea. We also downloaded all 8 available mitogenomes for the sister order Mantodea and 5 mitogenomes of Orthoptera used to root the tree.

Mitochondrial phylogenomics of Blattodea was performed based on the concatenation of all 13 protein-coding genes (Figure 1). Our results identified most clades as correctly assigned and monophyletic. An exception in the level of genus was found for *Periplaneta*, although it has been reported earlier (Cheng et al. 2016). The outgroups from Orthoptera clade were recovered with high bootstrap confidence, as well as most higher clades, such as Mantodea, Blattodea, Blaberoidea, Blattoidae, Termitoidae and Cryptocertoidae.

**Figure 1.**
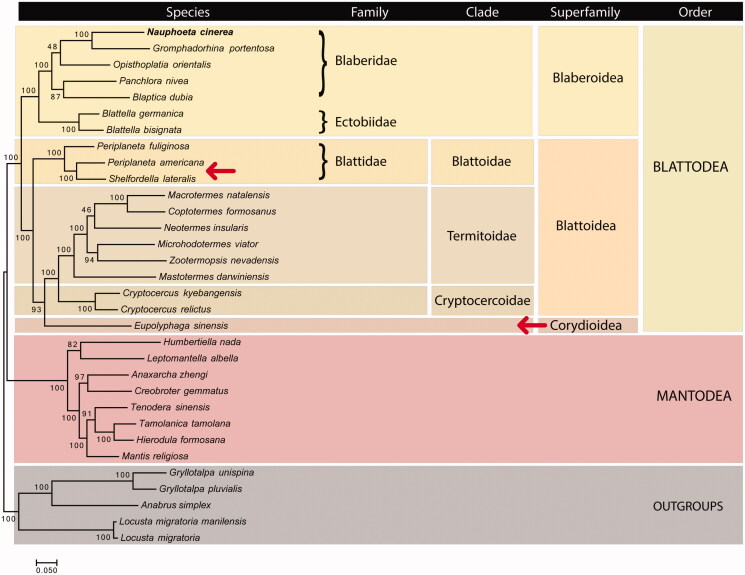
Mitochondrial phylogenomics of Blattodea and outgroups using a supermatrix approach on maximum likelihood. Bootstrap values for 100 replicates are shown at the corresponding nodes. The dataset was produced using phyloMito software (https://github.com/igorrcosta/phylomito). The accession numbers for sequences used here were: KY212743, NC_030001, NC_030002, NC_029224, NC_029225, NC_012901, NC_018549, NC_030003, NC_016951, NC_006076, NC_018120, NC_018122, NC_018124, NC_024658, NC_025522, NC_015800, NC_030191, NC_018132, NC_014274, NC_029326, NC_030261, NC_030261, NC_007702, NC_024028, NC_030264, NC_030267, NC_030268, NC_001712, NC_014891, NC_029148, NC_011302, NC_009967. The arrows indicate clades with putative problems in the systematic classification.

However, we did not find monophyly for the Blattoidea superfamily, mainly because of the position of *Eupolyphaga sinensis*. Our results indicate that Corydioidea should be seen as sister group to Termidoidae/Cryptocercoidae, being closer to species from those clades than Blattoidae species. Thus, Blattoidea turns to be polyphyletic (excluding *E. sinensis*) and should be disregarded as a *bona fide* systematic term. Considering the high support of *E. sinensis* relationship with Termitoidae and Cryptocercoidae (bootstrap = 93), we suggest reclassification of Corydiodea not as a superfamily but as a clade inside Blattoidea. Nuclear markers and further mitogenomes for Corydiodea species should be studied to confirm this relationship.
